# Comparing laser vs mechanical lithotripsy in suction mini‐PCNL for kidney stone disease: A prospective multicentre study by the endourology section of EAU

**DOI:** 10.1002/bco2.70075

**Published:** 2025-10-08

**Authors:** Angelo Cormio, Vineet Gauhar, Bhaskar K. Somani, Jaisukh Kalathia, Nariman Gadzhiev, Marek Zawadzki, Mahmoud Laymon, Karl Tan, Gopal Ramdas Tak, Theodoros Tokas, Madhu Sudan Agrawal, Jean de la Rosette, Kremena Petkova, Kazumi Taguchi, Dmitriy Gorelov, Alexey G. Martov, Leonardo Gomes Lopes, Mehmet ilker Gökce, Wissam Kamal, Stefania Ferretti, Devang Desai, Yadgar Abduljabbar Shwani, Khi Yung Fong, Steffi Kar Kei Yuen, Andreas Skolarikos, Marcos Cepeda, Thomas R. W. Herrmann, Daniele Castellani

**Affiliations:** ^1^ Department of Urology Azienda Ospedaliero‐Universitaria delle Marche, Università Politecnica Delle Marche Ancona Italy; ^2^ European Association of Urology Section of Endourology Arnhem Netherlands; ^3^ Department of Urology Ng Teng Fong General Hospital Singapore Singapore; ^4^ Department of Urology University Hospital Southampton NHS Trust Southampton UK; ^5^ Fortune Urology Clinic, Botad Gujarat India; ^6^ Department of Urology Saint‐Petersburg State University Hospital Saint‐Petersburg Russia; ^7^ Urology Unit, St. Anna Hospital Piaseczno Poland; ^8^ Department of Urology, Urology and Nephrology Center Mansoura University Mansoura Egypt; ^9^ Department of Surgery, Section of Urology Veterans Memorial Medical Center Quezon City Republic of the Philippines; ^10^ Department of Urology Asian Institute of Nephrology and Urology Hyderabad Telangana India; ^11^ Department of Urology, Medical School, University General Hospital of Heraklion University of Crete Heraklion Greece; ^12^ Department of Urology Pushpanjali Hospital & Research Centre Uttar Pradesh India; ^13^ International School of Medicine Istanbul Medipol University Istanbul Turkiye; ^14^ Bashkir State Medical University Ufa Russia; ^15^ Department of Urology and Nephrology Military Medical Academy Sofia Bulgaria; ^16^ Department of Urology University of Alabama at Birmingham Birmingham AL USA; ^17^ Department of Urology Hospital Orizonti and Santa Casa de Belo Horizonte Minas Gerais Brazil; ^18^ Department of Urology Ankara University School of Medicine Ankara Turkey; ^19^ Urology Unit, King Fahd General Hospital Jeddah Saudi Arabia; ^20^ Department of Urology University of Modena and Reggio Emilia Modena Italy; ^21^ University of Queensland Queensland Australia; ^22^ Erbil teaching hospital, Kurdistan Iraq; ^23^ Yong Loo Lin School of Medicine National University of Singapore Singapore Singapore; ^24^ Department of Surgery, S.H. Ho Urology Centre The Chinese University of Hong Kong Hong Kong, China; ^25^ Second Department of Urology, Sismanogleio Hospital National and Kapodistrian University of Athens Athens Greece; ^26^ Department of Urology Hospital Universitario Río Hortega Valladolid Spain; ^27^ Department of Urology Kantonspital Frauenfeld, Spital Thurgau AG Frauenfeld Switzerland; ^28^ Division of Urology, Department of Surgical Sciences Stellenbosch University Western Cape South Africa; ^29^ Hannover Medical School Hannover Germany; ^30^ Urology Unit, Azienda Ospedaliero‐Universitaria delle Marche Ancona Italy

**Keywords:** Kidney calculi, Laser, Percutaneous nephrolithotomy, Sheath, Suction

## Abstract

**Objectives:**

To compare perioperative outcomes, complications and stone‐free rates (SFRs) between laser and non‐laser lithotripsy in suction‐assisted mini‐PCNL (SM‐PCNL).

**Subjects and Methods:**

This prospective multicentre study enrolled adults with normal kidneys undergoing SM‐PCNL (14–22 Fr) across 30 international centres (March–November 2024). Patients were divided into laser (Group 1) and non‐laser (Group 2) groups. Propensity score matching (2:1) was performed based on age, sex, Guy's score and patient position. Primary outcomes were complications and 30‐day SFR assessed by CT. Multivariable logistic regression identified predictors of complete stone clearance and complications.

**Results:**

After matching, 748 patients were analysed (Group 1: 448; Group 2: 300). Non‐laser devices were associated with shorter lithotripsy (12 vs 18 min, p < 0.001) and operative times (37 vs 45 min, p < 0.001) and higher SFR (intraoperative: 91.3% vs 80.7%; 30‐day: 87.7% vs 82.1%). However, transfusions (3.3% vs 0.2%), pelvic perforation and pleural injury (each 3.0%) were more common in Group 2. On multivariable analysis, single‐step dilation (OR 3.05) and sheath sizes of 16.5–18 Fr (OR 1.98) or 20–22 Fr (OR 2.72) were associated with higher odds of stone‐free status, while skin‐to‐stone distance >8 cm (OR 0.5) and combined fluoroscopy/ultrasound access (0.28) reduced this likelihood. Stone volume (OR 1.03), serial dilation with non‐metal dilators (OR 2.64) and combined fluoroscopy/ultrasound access (OR 2.11) were factors associated with higher odds of complications. The lithotripsy technology had no direct bearing on complications.

**Conclusions:**

Both laser and non‐laser lithotripsy are effective in SM‐PCNL. Non‐laser devices improve efficiency and lasers were preferentially used with 14–18 fr access tracts.

## 1 INTRODUCTION

Percutaneous nephrolithotomy (PCNL) is recommended as the first‐line treatment option for renal stones larger than 2 cm and complex calculi, as endorsed by international guidelines.^1^ Subsequent developments in the technique have demonstrated that miniaturization of the percutaneous tract is associated with a reduction in complications, most notably haemorrhagic events while maintaining stone‐free rates (SFRs) comparable to those achieved with standard PCNL.^2^ With growing clinical experience and procedural refinement, mini‐PCNL, characterized by a tract size of no more than 22 Ch, has gained widespread acceptance and is now employed for the management of renal stones across a broad range of sizes^3^ and in patients with obesity.^4^ Moreover, the introduction of suction‐assisted devices has improved stone clearance whilst simultaneously minimizing the risk of complications.^5^

With miniaturized access, the utility of lasers in Mini‐PCNL has increased; however, it remains unclear whether these can fully replace conventional energy devices, as a recent meta‐analysis of randomized trials showed that non‐laser lithotripsy had better perioperative outcomes and SFR compared to holmium laser lithotripsy.^6^

To the best of our knowledge, comparative data evaluating perioperative outcomes and SFR between lasers and non‐laser devices for lithotripsy in suction mini‐PCNL in a real‐world practice are lacking. Hence, this study aimed to compare laser versus non‐laser lithotripsy in suction mini‐PCNL procedures by investigating intraoperative and perioperative parameters, complication rates and SFR in a multicentre experience.

## 2 SUBJECTS AND METHODS

Suction mini‐PCNL procedure was performed using a single‐use or reusable suction access nephrostomy sheath (14–22 Fr) combined with any energy device for lithotripsy. Only adult patients with kidney stones were prospectively enrolled between March and November 2024 across 30 centres in 21 countries. Inclusion criteria were age ≥ 18 years, kidney stone(s) only, suction sheath 14–22 Fr, normal kidney anatomy and a 2 mm slice unenhanced computed tomography (CT) scan performed no more than 6–8 weeks before surgery and 30 days after the procedure. Exclusion criteria encompassed an anomalous kidney, children/adolescents, concomitant ureteral stone, no suspension of antiplatelet/anticoagulants and patients unable to consent. Patients with incomplete data were also excluded. The full study protocol of the Suction Technology Utility in Mini‐PCNL Study (STUMPS) registry was previously published.^7^ The following data were gathered: patients' baseline characteristics, stone features, operative techniques, lithotripsy modalities, surgical time, complications, residual fragments (RFs) and reoperation rates. Preoperative urine cultures were collected in all patients and infections treated with appropriate antibiotics based on antibiogram. The largest stone diameter was documented for single or multiple stones, and stone volume (for the largest stone in cases of multiple stones) was calculated using the ellipsoid formula (length*width*depth*π*0.167). The Guy's stone score was also determined.^8^ Antibiotic prophylaxis followed local guidelines, while anticoagulants/antiplatelets were stopped at least 3 days before surgery and resumed at the discretion of the treating physician. The choice of energy source, patient positioning, puncture modality, tract dilation method, access sheath size and exit strategy were at the respective surgeons' discretion based on their experience and available resources. Pain on the first postoperative day was evaluated using a 10‐point visual analogue scale (VAS; 1 = lowest pain). The Clavien score validated for PCNL was used to classify complications within 30 days of surgery.^9^ A low‐dose unenhanced CT scan with 2 mm cuts was performed at 30 days of surgery to evaluate RFs in the bone window. Stone‐free status (SFS) was categorized as follows:

- *Grade A*: No fragments (100% stone‐free), zero residual fragment (ZRF)
- *Grade B*: Single residual fragment ≤4 mm (relatively stone‐free)
- *Grade C*: Single residual fragment >4 mm or multiple fragments of any size

### 2.1 Statistical analysis

Continuous variables are reported using median and interquartile range, while categorical variables are described using absolute numbers and percentages. Patients were divided into two groups according to the modality of lithotripsy. Group 1 included patients who had laser lithotripsy, whilst Group 2 included those having mechanical lithotripsy. To compare both study arms, patient demographics, peri‐operative parameters and 30‐day outcomes were compared between the groups using the Fisher exact test or χ^2^ test for categorical parameters and the Mann–Whitney U test for continuous variables. Propensity score matching was used to reduce confounding in the statistical comparisons. Propensity scores were calculated using a logistic regression model, and a soft 2:1 nearest‐neighbour matching for age, sex, Guy's stone score and patient position. To ensure optimal matching of covariates, the calliper width was started at 0.2,^10^, ^11^ with the target of absolute standardized mean difference (ASMD) for all covariates of <0.1,^11^ being achieved at a calliper width of 0.0009. All statistical comparisons were then repeated for the matched cohort like the overall cohort. Finally, two a priori multivariable logistic regression analyses were performed in the matched population to evaluate factors associated with Grade A stone‐free status and overall complications. Besides the comparison of lasers versus mechanical energies, variables that have been suggested in previous literature to impact SFR and complications (stone volume, puncture modality, tract dilation method, type of lithotripsy, patient position and sheath size) were entered into a multivariable model to assess their significance as independent predictors. These predictors are described using odds ratio (OR), 95% confidence interval (CI) and p‐values. All statistical analyses were performed using R Statistical language, version 4.3.0 (R Foundation for Statistical Computing, Vienna, Austria) with p < 0.05 indicating statistical significance.

## 3 RESULTS

**FIGURE 1:**
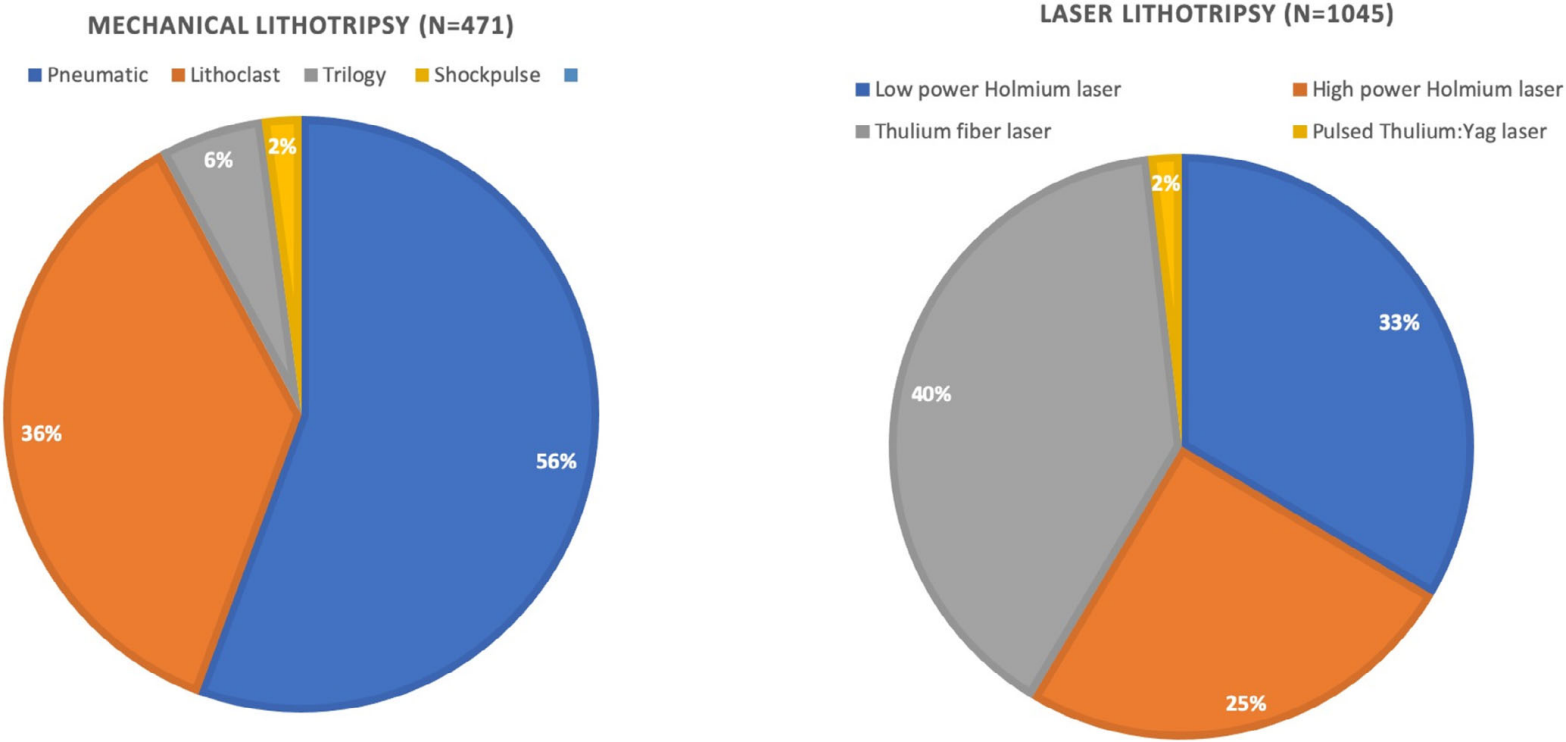
Proportion of lithotripsy modalities in the unmatched cohort.

**FIGURE 2:**
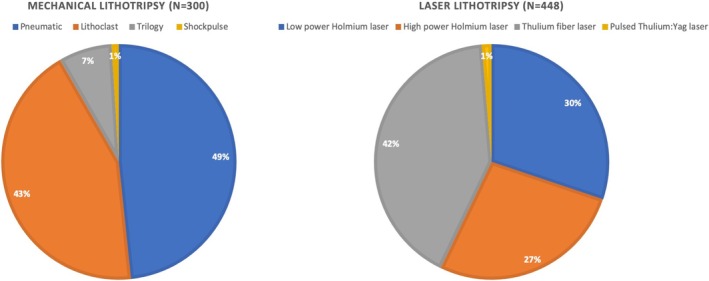
Proportion of lithotripsy modalities in the matched cohort.

A total of 1716 patients were included, with 1045 undergoing laser lithotripsy (Group 1) and 671 undergoing mechanical lithotripsy (Group 2). Table 1 shows patients' baseline characteristics before and after matching. After matching, 448 patients remained in Group 1 and 300 in Group 2. The median age was similar between groups (49 [41–58] years in group 1 vs 49 [40, 58] years in group 2, ASMD 0.02). The proportion of male patients was also comparable (Group 1: 37.1%, Group 2: 36.3%, ASMD 0.01). Demographically, groups were well‐balanced post‐matching for incidence of positive preoperative urine culture, stone volume, largest stone diameter, Guy's score and proportion of patients who had a previous PCNL on the same kidney (all covariates were matched to an ASMD of <0.1). There was a significant difference in body mass index, American Society of Anaesthesiologists score, Hounsfield unit and stone location. Table 2 shows procedural characteristics. In the matched population, a significantly higher proportion of patients in Group 1 had surgery under spinal anaesthesia (50.7% vs 40.7%, p = 0.01) but there was no difference in patient positioning. Significant differences in access technique were noted with higher fluoroscopy‐only access to the pelvicalyceal system in 79.3% of cases in Group 2 versus 68.8% in Group 1. Most of the patients in both groups had a single access tract (94.6% in group 1 vs 94.4% in group 2) with a similar need for a supracostal approach (24.3% in group 1 vs 30.3% in group 2, p = 0.08). Serial dilatation with metal dilatators was the preferred way to tract dilatation in Group 2 (48.6%), whilst single‐step dilatation was more common in the supine group (60.3%). A 14–16 Fr tract size was higher in Group 1 (71.9%), whilst 20–22 Fr access was more common in Group 2 (61.0%). Figures 1 and 2 show energy used for lithotripsy in unmatched and matched populations, respectively. The most common laser employed in the matched population was Thulium fibre laser (42.0%), while pneumatic lithotripsy was the most common energy in the non‐laser group (49%). The need for an additional basket for fragment evacuation did not differ between the groups (22.5% in group 1 vs 21.3% in group 2, p = 0.76). Lithotripsy time was significantly shorter in the non‐laser group (12 [6.0, 22] minutes vs 18 [11, 30] minutes, p < 0.001). Total surgical time was also shorter in Group 2 patients (37 [28, 60] minutes vs 45 [30, 75] minutes, p = 0.01). A tubeless procedure with a ureteral stent was the most common exit strategy in Group 1 (58.9%), while a nephrostomy tube with a ureteral stent was the most used in Group 2 (62.7%). A totally tubeless procedure was performed in 1.3% and 2% of cases in Group 1 and Group 2, respectively. Zero fragment intraoperative stone‐free status by fluoroscopy and/or visual inspection was high and seen in 91.3% of cases in Group 2 and 80.7% in Group 1. Table 3 shows intraoperative and postoperative outcomes. More than 80% of patients in both groups had no intraoperative bleeding following tract dilatation. However, there was a significant difference in blood transfusion rate (Clavien grade 2), with 10 cases (3.3%) in Group 2 versus only one case (0.2%) in Group 1 (p = 0.01). Only one patient needed angioembolization to control bleeding in Group 2 (Clavien grade 3b). Renal pelvic perforation managed by prolonged nephrostomy tube or postoperative placement of nephrostomy (Clavien grade 3a) occurred only in 3.0% of the cases in Group 2 (p = 0.01). Pleural injury with pneumothorax requiring a chest tube (Clavien grade 3a) occurred in 9 (3.0%) patients in Group 2 only, whilst colonic injury needing conservative management (Clavien grade 3a) occurred in 2 (0.4%) patients in Group 1 only. There was no significant difference in infectious complications, with no cases of sepsis requiring intensive care unit admission (Clavien grade 4) reported in the matched population. Median post‐operative hospital stay did not differ significantly (2.5 [1.0, 4.0] days in group 1 vs 2.0 [2.0, 4.0] days in group 2, p = 0.20). All‐cause readmission rate within 72 hours of discharge was low and similar in both groups (1.3% in group 1 vs 1.0% in group 2, p = 0.94).

**TABLE 1:** Patient's baseline characteristics.

	Unmatched			Matched			
	Group 1 (N = 1045)	Group 2 (N = 671)	ASMD	Group 1 (N = 448)	Group 2 (N = 300)	ASMD
Age, years, median [IQR]	50 [39, 60]	51 [40, 60]	0.04	49 [41, 58]	49 [40, 58]	0.02
Male gender, n (%)	422 (40.4)	201 (42.7)	0.05	166 (37.1)	109 (36.3)	0.01
ASA, n (%)			**0.13**			**0.16**
1	533 (51.0)	255 (54.1)		250 (55.8)	170 (56.7)	
2	405 (38.8)	177 (37.6)		160 (35.7)	114 (38.0)	
3	101 (9.7)	39 (8.3)		35 (7.8)	16 (5.3)	
4	6 (0.5)	0		3 (0.7)	0	
Body Mass Index, kg/m^2^, median [IQR]	27.0 [24.0, 30.0]	25.0 [22.5, 28.0]	**0.37**	27.0 [24.0, 29.9]	25.0 [22.6, 28.0]	**0.32**
Diabetes, n (%)	187 (17.9)	108 (22.9)	**0.12**	70 (15.6)	60 (20.0)	**0.11**
Chronic Kidney Disease, n (%)	233 (22.3)	74 (15.7)	**0.17**	83 (18.5)	44 (14.7)	**0.10**
Anticoagulant/antiplatelet use, n (%)	136 (13.0)	49 (10.4)	0.08	47 (10.5)	26 (8.7)	0.06
Presentation, n (%)			**0.30**			**0.17**
Hematuria	90 (8.6)	20 (4.2)		25 (5.6)	14 (4.7)	
Pain	739 (70.7)	377 (80.0)		337 (75.2)	242 (80.6)	
Fever	44 (4.2)	30 (6.4)		19 (4.2)	15 (5.0)	
Incidental	172 (16.5)	44 (9.4)		67 (15.0)	29 (9.7)	
First time stone former, n (%)	753 (72.1)	377 (80.0)	**0.19**	322 (71.9)	247 (82.3)	**0.25**
Preoperative positive urine culture, n (%)	203 (19.4)	67 (14.2)	**0.14**	82 (18.3)	45 (15.0)	0.09
Laterality, n (%)			**0.17**			**0.22**
Left	497 (47.6)	238 (50.5)		215 (48.0)	150 (50.0)	
Right	484 (46.3)	220 (46.7)		204 (45.5)	144 (48.0)	
Bilateral	64 (6.1)	13 (2.8)		29 (6.5)	6 (2.0)	
Guy's stone score, n (%)			0.09			0.07
1	552 (52.8)	251 (53.3)		281 (62.7)	178 (59.3)	
2	298 (28.5)	140 (29.7)		118 (26.4)	88 (29.4)	
3	132 (12.7)	61 (12.9)		48 (10.7)	33 (11.0)	
4	63 (6.0)	19 (4.1)		1 (0.2)	1 (0.3)	
Hounsfield units, median [IQR]	1120 [890, 1350]	1234 [1044, 1399]	**0.35**	1123 [900, 1349]	1230 [1044, 1400]	**0.32**
Largest stone diameter, cm, median [IQR]	2.0 [1.5, 2.8]	2.0 [1.6, 2.6]	0.02	1.9 [1.5, 2.7]	2.0 [1.7, 2.5]	0.04
Stone volume, mm^3^, median [IQR]	1541 [784, 2890]	1913 [1200, 4100]	0.09	1515 [770, 2935]	1877 [1155, 3845]	0.06
Stone location, n (%)						
Upper pole	133 (12.7)	73 (15.5)	**0.28**	49 (12.5)	45 (16.5)	**0.30**
Middle pole/pelvis	344 (32.9)	220 (46.7)		170 (43.4)	146 (53.7)	
Lower pole	376 (36.0)	132 (28.0)		173 (44.1)	81 (29.8)	
Multiple	192 (18.4)	46 (9.8)				
Previous PCNL on the same kidney, n (%)	36 (3.4)	13 (2.8)	0.04	12 (2.7)	6 (2.0)	0.04

**PCNL**: percutaneous nephrolithotripsy.

**ASMD**: absolute standardized mean difference.

**IQR:** interquartile range.

**Group 1**: laser lithotripsy.

**Group 2**: mechanical lithotripsy.

**TABLE 2:** Procedural characteristics.

	Unmatched			Matched		
	Group 1 (N = 1045)	Group 2 (N = 471)	p	Group 1 (N = 448)	Group 2 (N = 300)	p
Spinal anaesthesia, n (%)	489 (46.8)	209 (44.4)	0.41	227 (50.7)	122 (40.7)	**0.01**
Supine positioning, n (%)	666 (63.7)	200 (42.5)	**<0.001**	268 (59.8)	167 (55.7)	0.29
Puncture modality, n (%)			**<0.001**			**0.02**
Fluoroscopy only	696 (66.6)	389 (82.6)		308 (68.8)	238 (79.3)	
Ultrasound only	33 (3.2)	7 (1.5)		14 (3.1)	7 (2.3)	
Fluoroscopy and ultrasound	298 (28.5)	68 (14.4)		116 (25.9)	51 (17.1)	
Endoscopy guided	18 (1.7)	7 (1.5)		10 (2.2)	4 (1.3)	
Stone‐to‐skin distance >8 cm, n (%)	540 (51.7)	204 (43.3)	**0.01**	231 (51.6)	125 (41.7)	**0.01**
Number of tracts, n (%)			0.30			0.39
1	978 (93.6)	435 (92.4)		424 (94.6)	283 (94.4)	
2	62 (5.9)	34 (7.2)		24 (5.4)	15 (5.0)	
3	5 (0.5)	1 (0.2)		0	1 (0.3)	
4	0	1 (0.2)		0	1 (0.3)	
Supracostal access (above 11th rib), n (%)	254 (24.3)	178 (37.8)	**<0.001**	109 (24.3)	91 (30.3)	0.08
Tract dilation method, n (%)			**<0.001**			**<0.001**
Serial with metal dilators	217 (20.8)	238 (50.5)		119 (26.6)	146 (48.6)	
Serial with non‐metal dilators	108 (10.3)	58 (12.3)		45 (10.0)	24 (8.0)	
Balloon	31 (3.0)	9 (1.9)		14 (3.1)	2 (0.7)	
Single step dilatation	689 (65.9)	166 (35.2)		270 (60.3)	128 (42.7)	
Safety wire inserted during surgery, n (%)	420 (40.2)	181 (38.4)	0.55	176 (39.3)	134 (44.7)	0.16
Sheath size, n (%)			**<0.001**			**<0.001**
14–16 Fr	687 (65.7)	81 (17.2)		322 (71.9)	56 (18.7)	
16.5–18 Fr	286 (27.4)	98 (20.8)		92 (20.5)	61 (20.3)	
20–22 Fr	72 (6.9)	292 (62.0)		34 (7.6)	183 (61.0)	
Sheath brand, n (%)			**<0.001**			**<0.001**
Clearpetra	369 (35.3)	49 (10.4)		170 (37.9)	33 (11.0)	
Shah	299 (28.6)	202 (42.9)		135 (30.2)	153 (51.0)	
Others	377 (36.1)	220 (46.7)		143 (31.9)	114 (38.0)	
Lithotripsy modality, n (%)			**<0.001**			**<0.001**
Fragmentation only	486 (46.7)	353 (98.3)		202 (45.2)	225 (98.3)	
Dusting only	63 (6.1)	0		27 (6.0)	0	
Popcorning only	1 (0.1)	0		1 (0.2)	0	
Combination	489 (47.1)	6 (1.7)		217 (48.6)	4 (1.7)	
Basket required for stone extraction, n (%)	226 (21.6)	100 (21.2)	0.92	101 (22.5)	64 (21.3)	0.76
Lithotripsy time, min, median [IQR]	19 [12, 31]	12 [6.0, 25]	**<0.001**	18 [11, 30]	12 [6.0, 22]	**<0.001**
Total operation time, min, median [IQR]	48 [30, 79]	40 [29, 61]	**<0.001**	45 [30, 75]	37 [28, 60]	**0.01**
Intraoperative SFR by surgeon confirmed by fluoroscopy and visual inspection, n (%)			**0.01**			**<0.001**
100% clear	827 (79.3)	411 (87.3)		361 (80.7)	274 (91.3)	
Only dust remains	164 (15.7)	48 (10.2)		74 (16.6)	24 (8.0)	
Fragments remain	52 (5.0)	12 (2.5)		12 (2.7)	2 (0.7)	
Exit strategy, n (%)			**<0.001**			**<0.001**
Nephrostomy tube only	189 (18.1)	49 (10.4)		86 (19.3)	23 (7.7)	
Tubeless with stent	614 (58.8)	140 (29.7)		264 (58.9)	79 (26.3)	
Tubeless with overnight ureteric catheter	113 (10.8)	14 (3.0)		39 (8.7)	4 (1.3)	
Nephrostomy tube and stent	117 (11.2)	261 (55.4)		53 (11.8)	188 (62.7)	
Totally tubeless	12 (1.1)	7 (1.5)		6 (1.3)	6 (2.0)	
Tract closure modality, n (%)			**<0.001**			**<0.001**
No stitch	393 (37.6)	289 (61.4)		155 (34.6)	179 (59.7)	
Stitch placed	632 (60.5)	182 (38.6)		289 (64.5)	121 (40.3)	
Hemostatic agent	20 (1.9)	0		4 (0.9)	0	

**Group 1**: laser lithotripsy.

**Group 2**: mechanical lithotripsy.

**IQR:** interquartile range.

**TABLE 3:** Intra and postoperative outcomes.

	Unmatched			Matched		
	Group 1 (N = 1045)	Group 2 (N = 471)	p	Group 1 (N = 448)	Group 2 (N = 300)	p
Intraoperative bleeding after dilatation, n (%)			**<0.001**			**0.01**
No bleeding after tract dilatation	913 (87.4)	381 (80.9)		400 (89.3)	245 (81.7)	
Oozing partially obscuring vision despite suction but allowing surgery to continue	50 (4.7)	13 (2.8)		21 (4.7)	11 (3.7)	
Suction helps keep vision clear	77 (7.4)	77 (16.3)		26 (5.8)	44 (14.7)	
Case abandoned	5 (0.5)	0		1 (0.2)	0	
Blood transfusion (Clavien 2)	1 (0.1)	16 (3.4)	**<0.001**	1 (0.2)	10 (3.3)	**0.01**
Bleeding managed by angioembolisation (Clavien 3b)	0	1 (0.2)	0.68	0	1 (0.3)	0.84
Renal pelvic perforation managed by prolonged nephrostomy tube or postoperative placement of nephrostomy (Clavien 3a)	4 (0.4)	16 (3.4)	**<0.001**	0	9 (3.0)	**0.01**
Colon perforation managed conservatively (Clavien 3a)	2 (0.2)	0	0.853	2 (0.4)	0	0.66
Pneumothorax managed by intercostal draining under local anaesthesia (Clavien 3a), n (%)	0	10 (2.1)	**<0.001**	0	3 (1.0)	0.13
Postoperative pain score, median [IQR]	2.0 [1.0, 3.0]	2.0 [1.0, 4.0]	0.18	2.0 [1.0, 3.0]	2.0 [1.0, 4.0]	**0.01**
Infectious complications, n (%)			0.67			0.36
None	970 (92.8)	431 (91.5)		417 (93.1)	285 (95.0)	
Symptomatic UTI managed using antibiotics (Clavien 2)	73 (7.0)	39 (8.3)		31 (6.9)	15 (5.0)	
Urosepsis with multiple organ failure requiring ICU management (Clavien 4)	2 (0.2)	1 (0.2)		0	0	
Bleeding managed using IV fluid without need for blood transfusion (Clavien 1)	7 (0.7)	2 (0.4)	0.83	3 (0.7)	1 (0.3)	0.91
Postoperative RF grade on 30‐day CT scan, n (%)			0.18			0.10
Grade A: zero RF	859 (82.2)	405 (86.0)		368 (82.1)	263 (87.7)	
Grade B: single RF ≤ 4 mm	132 (12.6)	48 (10.2)		65 (14.6)	28 (9.3)	
Grade C: single RF > 4 mm/multiple of any size	54 (5.2)	18 (3.8)		15 (3.3)	9 (3.0)	
Hospital stay, days, median [IQR]	3.0 [2.0, 3.0]	2.0 [2.0, 3.0]	0.90	2.5 [1.0, 4.0]	2.0 [2.0, 4.0]	0.20
Readmission for any reason within 72 hours, n (%)	19 (1.8)	6 (1.3)	0.58	6 (1.3)	3 (1.0)	0.94
Reintervention after 30 days, n (%)	35 (3.3)	1 (0.2)	**<0.001**	10 (2.2)	1 (0.3)	0.07

**Group 1**: laser lithotripsy.

**Group 2**: mechanical lithotripsy.

**ICU:** intensive care unit.

**IQR:** interquartile range.

**UTI:** urinary tract infection.

**RF**: residual fragment.

At the 30‐day CT scan evaluation, overall SFR was similar, and Grade A stone‐free status was reached in 82.1% and 87.7% of cases in the laser and non‐laser groups, respectively. Reintervention for RFs was planned in 2.2% of the patients in group 1 and 0.3% in group 2 (p = 0.32).

At multivariable analysis (Table 4), fluoroscopy and ultrasound puncture (OR 0.28, 95% CI 0.15–0.59, p < 0.001), and skin to stone distance >8 cm (OR 0.51 95% CI 0.32–0.79, p = 0.01) were factors significantly associated with lower odds of being Grade A stone‐free, whilst single step tract dilatation (OR 3.05, 95% CI 1.65–5.73), sheath size 16.5‐18 Fr (OR 1.98, 95% CI 1.09–3.70, p = 0.03) and sheath size 20‐22 Fr (OR 2.72,95% CI 1.30–588, p = 0.01) were factors associated with higher odds of being Grade A stone‐free. Stone volume (OR 1.03, 95% CI 1.00–1.07, p = 0.02), serial tract dilatation with non‐metal dilators (OR 2.64, 95% CI 1.06–6.46, p = 0.03) and fluoroscopy and ultrasound puncture (OR 2.11, 95% CI 1.08–4.13, p = 0.03) were factors significantly associated with higher odds of all complications (Table 4).

**TABLE 4:** Multivariable regression analysis of factors affecting grade A stone‐free status and overall complications.

	Grade A stone‐free status		All complications	
	*OR (95% CI)*	*p*	*OR (95% CI)*	*p*
Mechanical lithotripsy (reference laser)	1.05 (0.62–1.82)	0.85	1.19 (0.64–2.18)	0.58
Supine (reference prone)	1.12 (0.68–1.85)	0.65	0.66 (0.37–1.19)	0.17
Stone volume	0.98 (0.95–1.01)	0.20	1.03 (1.00–1.07)	**0.02**
Puncture modality (reference fluoroscopy only)				
Ultrasound only	0.35 (0.12–1.17)	0.06	0 (0–53)	0.98
Fluoroscopy and ultrasound	0.28 (0.15–0.49)	**<0.001**	2.11 (1.08–4.13)	**0.03**
Endoscopy guided	1.74 (0.32–32.65)	0.60	1.11 (0.06–6.26)	0.92
Skin to stone distance >8 cm (vs ≤ 8 cm)	0.51 (0.32–0.79)	**0.01**	0.99 (0.59–1.67)	0.98
Tract dilation method (reference serial with metal dilators)				
Serial with non‐metal dilators	0.5 (0.25–1.01)	0.05	2.64 (1.06–6.46)	**0.03**
Balloon	2.34 (0.40–45.01)	0.44	1.92 (0.38–7.37)	0.38
Single‐step dilatation	3.05 (1.65–5.73)	**<0.001**	1.04 (0.52–2.10)	0.92
Sheath size (reference 12–14 Fr)				
16.5–18 Fr	1.98 (1.09–3.70)	**0.03**	1.25 (0.60–2.53)	0.55
20–22 Fr	2.72 (1.30–5.88)	**0.01**	1.9 (0.88–4.16)	0.10

## 4 DISCUSSION

A variety of lithotripters have been developed using ballistic, ultrasonic and laser energy to fragment stones.^12^ As technology advances, modern lasers for lithotripsy in min‐PCNL have gained popularity, but their performance vis a vis conventional and newer non‐laser devices, like trilogy and ShockPulse, is yet to be ascertained in a real‐world setting, especially when used with different size equipment in performing SM‐PCNL. To the best of our knowledge, this analysis from the STUMPS registry provides the first real‐world comparison of laser versus non‐laser lithotripsy in SM‐PCNL. Non‐laser energy sources were associated with shorter lithotripsy and overall operative times, along with marginally higher intraoperative and 30‐day SFRs. However, these benefits were accompanied by a higher incidence of bleeding‐related and visceral complications, such as transfusions, renal pelvic perforation and pleural injury. Indeed, the tract size was larger in this cohort, possibly contributing to renal access trauma. We postulate that the semirigid device probes better suited for larger tract access may in themselves cause mucosal injury, iatrogenic trauma to the collecting system, or restrict sheath movement, which might unknowingly cause more parenchymal damage while attempting to manipulate the sheath in the pelvic caliceal system. Whilst not confirmed, past studies comparing these devices in standard PCNL have been controversial, with differences in safety profile and surgeon satisfaction being reported, especially related to possible parenchymal injury.^13^, ^14^

Previous studies comparing pneumatic and ShockPulse devices with Holmium:YAG laser similarly reported faster fragmentation efficiency but higher complication rates for non‐laser technologies.^15^, ^16^ In addition, York et al. found no significant differences in adjusted SFR or complications across three lithotripters, namely a dual‐probe ultrasonic device, a combination pneumatic and ultrasonic device, and a pneumatic device, reinforcing the concept that energy modality alone does not define clinical outcomes.^12^

Our results are consistent with a recent meta‐analysis showing shorter operative times and better SFR with non‐laser energy compared to Holmium:YAG laser in standard PCNL.^6^ Despite high‐power lasers with adjustable pulse energy and frequency settings significantly enhancing the efficiency and speed of stone fragmentation that can be easily aspirated or flushed out, potentially eliminating the need for fragment retrieval in flexible ureteroscopy and thereby reducing both operative time and residual stone fragments, they may not be as suitable for mini‐PCNL procedures, as demonstrated by our findings of shorter operative time in the non‐laser group and comparable SFR.

However, our study extends this comparison to suction‐assisted mini‐PCNL and includes modern laser platforms, particularly Thulium fibre laser (TFL), which has better ablation efficiency and enhanced thermal control, both useful properties especially when managing large stone burdens.^17^, ^18^ Patil et al. compared TFL and dual‐energy pneumatic‐ultrasonic systems such as Trilogy™ in suction‐assisted mini‐PCNL. While Trilogy™ achieved significantly faster fragmentation rates, TFL showed comparable safety outcomes and reached complete stone clearance at one‐month follow‐up.^19^ Large et al.^20^ demonstrated that Trilogy™ outperformed ShockPulse‐SE regarding clearance speed and reliability, despite similar final SFRs, highlighting that device performance can vary even within non‐laser technologies. While lithotripsy energy sources may not significantly affect SFR, they do influence complications and overall outcomes.^12^, ^15^, ^16^, ^19^ Conversely, our results show that energy modality was not independently associated with complications in the multivariable analysis. Surgeons should tailor energy selection based on stone volume, anatomy and personal experience, balancing operative time against complication risk in complex cases. PCNL results depend on a combination of factors, including stone burden, access technique, sheath selection and overall procedural strategy. This is even more pertinent when using suction M‐PCNL, where there is a high flow environment due to fluid inflow and aspiration occurring simultaneously, and this can cause differential distension and collapse of the pelvicalyceal system depending on the tract size. Not only pneumatic or ballistic devices but indeed lasers too, especially pulse modulated and high power lasers, may induce thermal damage and potential mucosal bleeding if used carelessly or inadvertently. Caution must be exercised on how to manage the intraoperative handling of any lithotripsy modality for a safe and efficacious procedure whilst simultaneously doing suction aspiration. We are limited as surgeons in our study did not report the size of the probes or laser fibres used and the settings or preferred technique of lithotripsy. Yet, in a real‐world practice, this variability and personal preference bias are expected as surgeons tailor and continuously modify strategies as the kidney stone is progressively managed. Even the fluid management strategy may play a role, and we are also limited by the lack of this information. In our study, none of the surgeons reported the need or preference for using intra renal pressure monitoring as it is well‐established that suction PCNL lowers the intrarenal pressure.^5^ This, alongside good antibiotic stewardship, helped prevent any serious septic complications in our study.

Beyond the lithotripsy modality, technical factors had also a significant impact. In our multivariable analysis, single‐step tract dilation and larger sheaths were independently associated with improved SFR. This is in line with a recent study showing that one‐step dilation significantly shortens access and fluoroscopy times compared to serial dilation, without increasing the risk of bleeding or complications.^21^ In contrast, combined fluoroscopy and ultrasound‐guided puncture, although often employed for precision, was paradoxically linked to lower SFR and higher complication rates in our cohort. This likely reflects underlying procedural complexity, as the combined use of fluoroscopy and ultrasound is often adopted in challenging anatomies.^22^ While Corrales et al. reported similar outcomes between ultrasound‐ and fluoroscopy‐guided access, their findings were limited to single‐modality use.^22^ In contrast, our results suggest that dual‐modality access may be associated with more complex cases and lower SFR. These findings underscore that outcomes in suction mini‐PCNL are multifactorial and that refining surgical technique is as important as selecting the optimal lithotripsy energy source.

Our study is not devoid of limitations. The STUMPS registry's international real‐world multicentre practice dataset is its strength, allowing for a match paired analysis in over 700 cases from 30 centres in 21 countries. This enhances the generalizability of findings, but it also highlights how variability in surgeon expertise, resources, institutional practices and patient management strategies do affect outcomes in different hospital practices, yet this in itself may represent a source of bias. For example, spinal anaesthesia was more common in the laser group, while metallic dilators were predominant in the non‐laser group, potentially reflecting possible institutional or surgeon choice in how cases are approached. Cost and accessibility, which were beyond the scope of this paper, are additional considerations. New lasers like TFL and pulsed Thulium:Yag laser are not universally available, especially in low‐resource settings, where pneumatic and ultrasonic devices remain more affordable, and this will induce a surgeons preferential bias to choose the same. Future research should account for these factors and could focus on how to harmonize the same. Our study is also limited by its observational nature and possible confounding factors despite propensity score matching. Procedural details like suction or irrigation pressure were not standardized, and the choice of energy device was left to the surgeon's discretion, which improves real‐world relevance but limits standardization. Additionally, we pooled all lasers against all non‐laser devices, and hence we cannot recommend specific device strategies for individualized approach to different stone burdens.

While non‐laser devices may optimize surgical time, lasers were associated with lower rates of bleeding complications and visceral injuries, suggesting improved safety in terms of reduced collateral tissue damage. Both patient characteristics and institutional resources must guide decision‐making. These findings from the STUMPS registry reinforce the universality of suction‐assisted mini‐PCNL, which is performed globally using various energy sources depending on availability. Our results suggest that the energy device acts as a complementary tool to enhance lithotripsy efficiency across different sheath sizes. We feel that the knowledge here can help design specific trials to see how to match stone characteristics with laser and no laser devices when using different size access tracts in suction M‐PCNL. Ideally, this would better help categorize specific recommendations. However, the energy milieu in endourology continues to evolve very fast, and its becoming harder to strategize. Hence, in our take home message from these observations we report that lasers are preferred in smaller tracts and non‐laser devices for larger access. This can definitely affect the outcomes depending on stone burden.

## 5 CONCLUSION

Urologists may safely use both laser and non‐laser lithotripsy devices while exercising necessary caution in access technique to mitigate mini‐PCNL‐related complications. As stone burden has a direct impact on operative time and SFR, pairing the right lithotripsy technology with access tract can help improve the same. In our study, non‐laser lithotripsy has shown an advantage for the same. But evidently, lasers are selectively preferred with smaller tracts.

## AUTHOR CONTRIBUTIONS

Study conception: Vineet Gauhar, Daniele Castellani and Angelo Cormio. Data collection: Vineet Gauhar, Jaisukh Kalathia, Nariman Gadzhiev, Marek Zawadzki, Mahmoud Laymon, Karl Tan, Gopal Ramdas Tak, Theodoros Tokas, Madhu Sudan Agrawal, Kremena Petkova, Kazumi Taguchi, Dmitriy Gorelov, Alexey G. Martov, Leonardo Gomes Lopes, Mehmet Ilker Gökce, Wissam Kamal, Stefania Ferretti, Devang Desai, Yadgar Abduljabbar Shwani, Steffi Kar‐Kei Yuen, and Marcos Cepeda. Statistical analysis: Khi Yung Fong and Daniele Castellani. Manuscript writing: Angelo Cormio, Daniele Castellani and Vineet Gauhar. Manuscript review: Stefania Ferretti, Thomas R.W. Herrmann, Andreas Skolarikos and Bhaskar K Somani. Clinical supervision: Jean de la Rosette and Vineet Gauhar.

## CONFLICT OF INTEREST STATEMENT

The authors declare no conflicts of interest.

## HUMAN RIGHTS

Institutional board review approval was obtained by the leading centre (Asian Institute of Nephro‐Urology, AINU #01/2024) and the remaining centres had approvals from their Institutional board.
